# Leaching Characteristics of Exogenous Cl^−^ in Rain-Fed Potato Fields and Residual Estimation Model Validation

**DOI:** 10.3390/plants14142171

**Published:** 2025-07-14

**Authors:** Jiaqi Li, Jingyi Li, Hao Sun, Xin Li, Lei Sun, Wei Li

**Affiliations:** 1 College of Resources and Environment, Northeast Agricultural University, Harbin 150030, China; s210201014@neau.edu.cn (J.L.); ljingyi0626@163.com (J.L.); sunhao@nenu.edu.cn (H.S.); swx05256lx@126.com (X.L.); 2 Key Laboratory of Germplasm Enhancement, Physiology and Ecology of Food Crops in Cold Region, Northeast Agricultural University, Ministry of Education, Harbin 150030, China; 3 National Key Laboratory of Smart Farm Technologies and Systems, Northeast Agricultural University, Harbin 150030, China

**Keywords:** Cl^−^ leaching, chloride-containing fertilizer, rain-fed agriculture, *Solanum tuberosum* L., Cl^−^ residual estimation model

## Abstract

Potato (*Solanum tuberosum* L.) is a chlorine-sensitive crop. When soil Cl^−^ concentrations exceed optimal thresholds, the yield and quality of potatoes are limited. Consequently, chloride-containing fertilizers are rarely used in actual agricultural production. Therefore, two years of field experiments under natural rainfall regimes with three chlorine application levels (37.5 kg ha^−1^/20 mg kg^−1^, 75 kg ha^−1^/40 mg kg^−1^, and 112.5 kg ha^−1^/60 mg kg^−1^) were conducted to investigate the leaching characteristics of Cl^−^ in field soils with two typical textures for Northeast China (loam and sandy loam soils). In this study, the reliability of Cl^−^ residual estimation models across different soil types was evaluated, providing critical references for safe chlorine-containing fertilizer application in rain-fed potato production systems in Northeast China. The results indicated that the leaching efficiency of Cl^−^ was significantly positively correlated with both the rainfall amount and the chlorine application rate (*p* < 0.01). The Cl^−^ migration rate in sandy loam soil was significantly greater than that in loam soil. However, the influence of soil texture on the Cl^−^ leaching efficiency was only observed at lower rainfall levels. When the rainfall level exceeded 270 mm, the Cl^−^ content in all the soil layers became independent of the rainfall amount, soil texture, and chlorine application rate. Under rain-fed conditions, KCl application at 80–250 kg ha^−1^ did not induce Cl^−^ accumulation in the primary potato root zone (15–30 cm), suggesting a low risk of toxicity. In loam soil, the safe application range for KCl was determined to be 115–164 kg ha^−1^, while in sandy loam soil, the safe KCl application range was 214–237 kg ha^−1^. Furthermore, a predictive model for estimating Cl^−^ residuals in loam and sandy loam soils was validated on the basis of rainfall amount, soil clay content, and chlorine application rate. The model validation results demonstrated an exceptional goodness-of-fit between the predicted and measured values, with R^2^ > 0.9 and NRMSE < 0.1, providing science-based recommendations for Cl-containing fertilizer application to chlorine-sensitive crops, supporting both agronomic performance and environmental sustainability in rain-fed systems.

## 1. Introduction

Chlorine (Cl), an essential nutrient for plants, is readily introduced into soil through exogenous pathways, such as rainfall, fertilization, and irrigation [1]. Cl exists exclusively in soil solution in a stable ionic form (Cl^−^). Owing to its high solubility in soil and weak adsorption into soil particles, the migration of Cl^−^ in soil is governed primarily by water flux [2,3]. The leaching loss of Cl^−^ is significantly positively correlated with the rainfall/irrigation volume. When the soil water input exceeds the evapotranspiration level, downward vertical migration occurs [4,5]. Furthermore, the residual Cl^−^ content varies among soils with different textures. In more loosely textured soils, Cl^−^ is more prone to leaching with water movement. Under identical Cl^−^ application rates and irrigation conditions, the residual Cl^−^ content is highest in clay loam, followed by loam, sandy loam, and finally sandy soil [6]. Determining the migration patterns of Cl^−^ in soils with various textures under natural rainfall regimes and developing corresponding predictive models are important for agricultural production, particularly for chlorine-sensitive crops.

Given the multiple sources of Cl^−^ in soils, chlorine deficiency rarely occurs in agricultural practice, whereas excess chlorine poses greater concerns for chlorine-sensitive crops. As a chlorine-sensitive crop, potato (*Solanum tuberosum* L.) requires the restricted application of chlorine-containing fertilizers. Studies have demonstrated that the application of chlorine-containing fertilizer in non-irrigated regions reduces both tuber yield and potato quality [7,8,9]. Under irrigated conditions, the application of chlorine-containing fertilizer facilitates Cl^−^ leaching into deeper soil layers, effectively increasing tuber yield and potato quality [10,11,12]. In practical potato cultivation, vegetation interception owing to natural rainfall may alter the Cl^−^ transport patterns relative to the patterns observed in simulation studies. The combined effects of soil texture variability and rainfall differentials on Cl^−^ migration within the potato root zone remain poorly quantified.

Previous studies on soil nutrient transport have focused primarily on either empirical or mechanistic models. These approaches typically involve equation development on the basis of observational data or investigations of physicochemical soil processes to predict nutrient dynamics, ultimately guiding the development of fertilization strategies for crop production [13,14]. In studies on Cl^−^ migration in soils, leaching efficiency is typically determined by measuring Cl^−^ transport to 1 m or deeper soil layers. However, this method may result in higher rainfall or irrigation amounts than actually required. Since potato roots are primarily distributed in the 15–30 cm soil layer, maintaining Cl^−^ within the safe soil threshold does not necessitate excessive rainfall or irrigation [15]. Moreover, most simulations have been conducted under bare soil conditions, potentially neglecting the interception of rainfall or irrigation by vegetation. This phenomenon may lead to the overestimation of the Cl^−^ leaching efficiency in soils relative to the real-world efficiency [16]. Consequently, it is unclear whether rainfall in rain-fed agricultural systems can effectively leach applied Cl^−^ from farmland soils and how soil texture differences affect leaching efficiency. The validity of existing models for quantifying Cl^−^ retention in loam and sandy loam soils solely on the basis of the rainfall/irrigation volume, soil clay content, and chlorine application rate must be further verified.

Suihua and Mudanjiang are major potato production regions in Northeast China. Despite concurrent rainfall and heat during the growing season, which involves extensive rainfall, KCl fertilizer application remains uncommon in rain-fed potato cultivation systems. Although S is an essential nutrient for plant growth, the soils in Suihua and Mudanjiang can provide sufficient sulfur for potato cultivation without exhibiting sulfur deficiency. Compared with KCl, K_2_SO_4_ is not only more expensive and contains lower potassium content but also has excessive SO_4_^2−^, which may induce oxidative stress that inhibits tuber formation and development in potatoes [17]. Therefore, elucidating the mechanisms by which rainfall and irrigation regulate the Cl^−^ distribution within the potato root zone across different soil textures can provide critical guidance for chlorine-containing fertilizer application in rain-fed potato production systems in Northeast China. To address these questions, field experiments were conducted in Suihua (loam soil) and Mudanjiang (sandy loam soil), and the soil Cl^−^ residual estimation model developed by Sun et al. [6] was utilized to investigate the following questions: (1) What are the migration and leaching characteristics of Cl^−^ in different textured soils during rain-fed potato cultivation? (2) Can Cl^−^ residual estimation models reliably predict the soil Cl^−^ content in rain-fed potato fields?

## 2. Results

### 2.1. Effect of Cl Application Rate on Cl^−^ Migration in Soil

**Figure 1:**
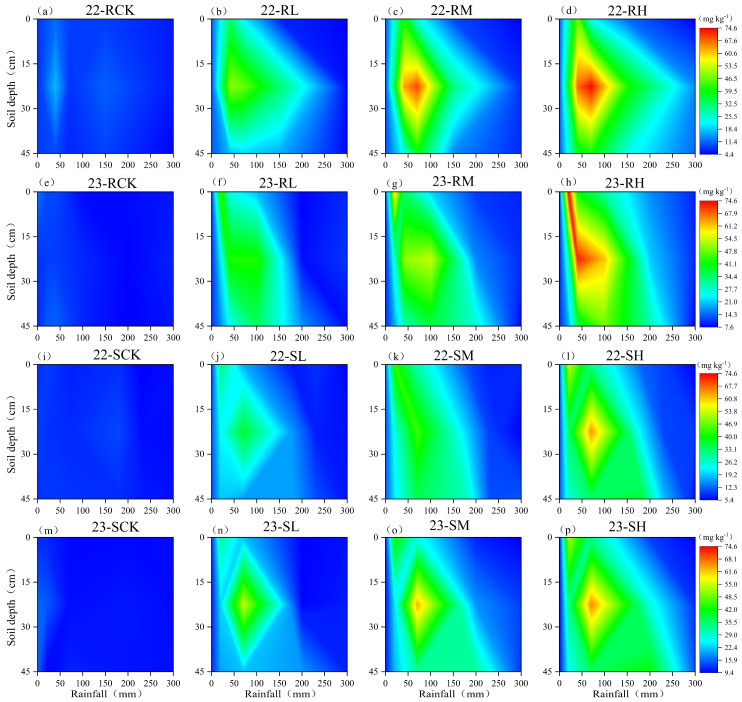
Dynamic migration patterns of Cl^−^ in loam soil (**a**–**h**) and sandy loam soil (**i**–**p**) under different rainfalls, showing temporal variations in soil Cl^−^ concentration at 15 cm, 30 cm, and 45 cm depths. Note: This visualization presents observed field data only. R—loam soil; S—sandy loam soil; L, M, H—low, medium, and high Cl application rate; CK—no Cl application.

**Figure 2:**
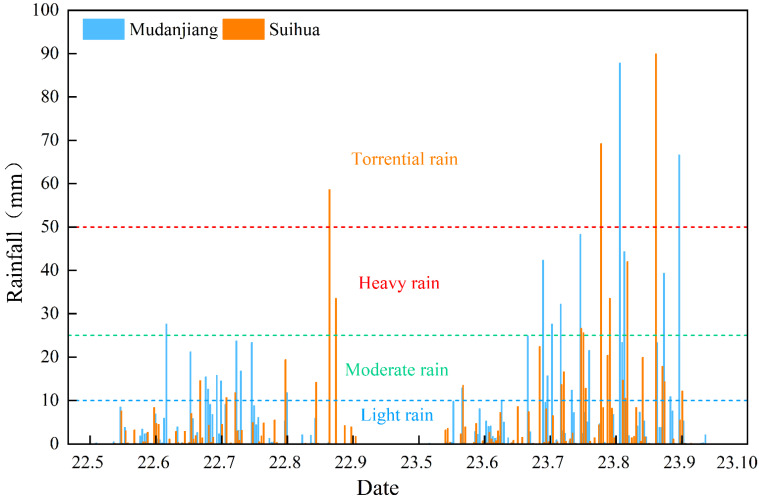
Rainfall statistics for potato growing season at experimental sites in 2022 and 2023.

The migration of Cl^−^ in soil was significantly affected by the Cl application rate (Figure 1). With decreasing rainfall, the Cl^−^ content in the fertilized soil layer increased significantly with increasing Cl application rates due to weak leaching (*p* < 0.05). With increasing rainfall, the Cl^−^ content in the fertilized soil layer began to migrate downward. The transport of Cl^−^ was concentrated in the sandy loam soil but trailed in the loam soil, and this phenomenon became more obvious with increasing Cl^−^ application rates. At the sandy loam test site, the amount of rainfall during the potato growth period in 2022 (340 mm) was significantly lower than that in 2023 (700 mm). However, because several heavy rainfall events (≥8 mm h^−1^) occurred in the early potato growth period in 2022, the migration of Cl^−^ in 2022 was faster than that in 2023, which was a year dominated by light rain (≤2.5 mm h^−1^) in the same period (Figure 2). With a rainfall level of approximately 270 mm and an application level of less than 250 kg KCl ha^−1^ in loam soil or sandy loam soil, more than 85% of the Cl^−^ introduced into the soil could be leached below the 45 cm soil layer. In addition, the residual Cl^−^ in the 0–45 cm soil layer was less than 20 mg kg^−1^. There was no significant difference in Cl^−^ content in the soils treated with and without Cl, whether in loam soil or sandy loam soil (*p* > 0.05). The influence of the Cl application rate on the soil Cl^−^ content was limited to only the chlorinated soil layer in periods with low rainfall, and the effect gradually disappeared with increasing rainfall.

**Figure 3:**
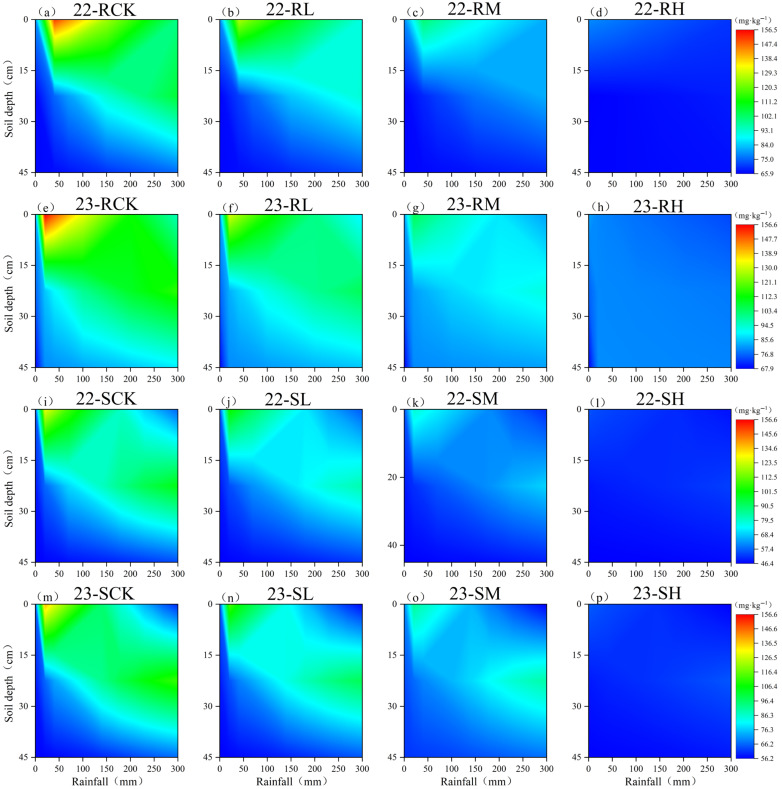
Dynamic migration patterns of SO_4_^2−^ in field loam soil (**a**–**h**) and sandy loam soil (**i**–**p**) under different rainfalls, showing temporal variations in soil Cl^−^ concentration at 15 cm, 30 cm, and 45 cm depths. Note: This visualization presents observed field data only. R—loam soil; S—sandy loam soil; L, M, H—low, medium, and high Cl application rate; CK—no Cl application.

The sulfate (SO_4_^2−^) content varied among different chloride (Cl^−^) application treatments, but its influence on the leaching process was significantly less pronounced compared to Cl^−^ (Figure 3). Similarly to Cl^−^, SO_4_^2−^ applied to the soil migrated downward with rainfall, but its migration rate was significantly lower (*p* < 0.05) than that of Cl^−^, and no obvious tailing phenomenon, as observed for Cl^−^, was detected. Furthermore, despite differences in SO_4_^2−^ application rates, the leaching rates among treatments did not show significant differences (*p* > 0.05). A comparison between loam and sandy loam soils revealed that the migration rate of SO_4_^2−^ was higher in sandy loam than in loam. When cumulative rainfall reached 300 mm, the difference in SO_4_^2−^ content between the 0–15 cm and 15–30 cm layers was not significant (*p* > 0.05) in loam, whereas it was significant (*p* < 0.05) in sandy loam. However, more than 45% of the applied SO_4_^2−^ remained within the 0–45 cm soil layer across all treatments. Additionally, although SO_4_^2−^ application rates differed among treatments, neither the cumulative leaching loss of Cl^−^ nor the rainfall required for Cl^−^ leaching exhibited statistically significant variations (*p* > 0.05). These findings suggest that SO_4_^2−^ migration is less dynamic than that of Cl^−^ and that varying SO_4_^2−^ application rates do not significantly alter Cl^−^ leaching behavior under the tested conditions.

### 2.2. Effect of Rainfall on Cl^−^ Distribution in Soil Profile

**Figure 4:**
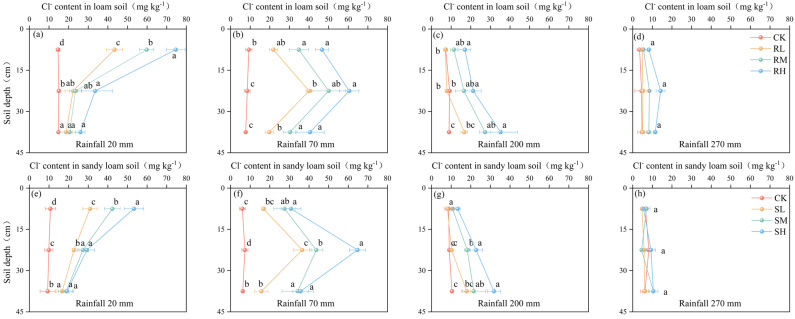
Spatial distribution of Cl^−^ in loam soil (**a**–**d**) and sandy loam soil (**e**–**h**) under different rainfall levels. Different rainfall amounts correspond to different growth stages of potatoes: 20 mm—pre—emergence stage; 70 mm—seedling and tuber initiation stage; 200 mm—tuber bulking stage; 270 mm—starch accumulation and harvest stage. R—loam soil; S—sandy loam soil; L, M, and H—low, medium, and high rates of Cl application, respectively. Different lowercase letters (a, b, c, d) indicate significant differences between Cl treatments (*p* < 0.05).

When the rainfall amount was 20 mm, Cl^−^ began to migrate from 0–15 cm to the lower soil layer in both the loam soil and sandy loam soil. However, the effect of rainfall on Cl^−^ leaching was very weak, and approximately 85% of the exogenous Cl^−^ was still distributed in the 0–15 cm soil layer, which was the soil layer in which fertilizer was applied (Figure 4). When the rainfall amount accumulated to 70 mm, approximately 41.0–49.1% of the Cl^−^ was distributed in the 15–30 cm soil layer for the loam soil, and 41.5–52.9% was distributed in the sandy loam soil. While there were essentially no differences between the values in the different soil types, the leaching effect of Cl^−^ with rainfall became increasingly obvious. When the Cl^−^ content in the 15–30 cm soil layer was significantly greater than that in the other two soil layers (*p* < 0.05), 200 mm of rainfall was necessary to observe the same effects in loam soil, whereas 150 mm of rainfall was necessary for sandy loam. In the rainfall range of 150–200 mm, the Cl^−^ content in the 0–30 cm soil layer of the loam soil was greater than that in the sandy loam soil for the same amount of rainfall, whereas the Cl^−^ content in the 30–45 cm soil layer was greater in the sandy loam soil. This finding indicated that the migration of Cl^−^ in the loam soil was slightly more difficult than that in the sandy loam soil. During the rainy season, the increased rainfall caused the Cl^−^ to migrate quickly to deeper soil layers. At a rainfall depth of 270 mm, there was no difference in the Cl^−^ content in the 0–45 cm soil layer in either the loam or sandy loam soil, regardless of the treatment type (*p* > 0.05).

### 2.3. Effects of Rainfall and Cl Application Rate on Cl^−^ Leaching Efficiency

**Figure 5:**
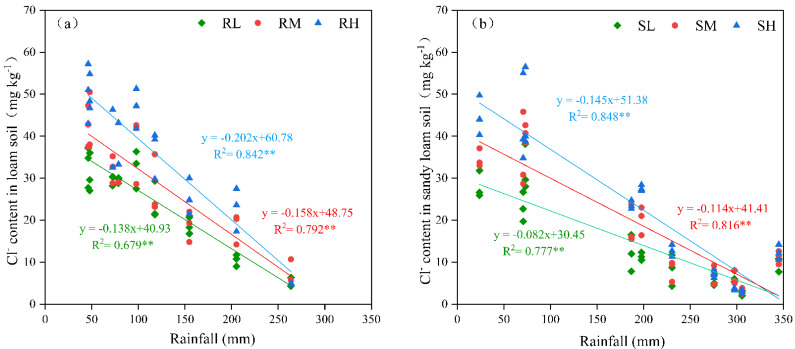
The effect of the Cl application rate on the Cl^−^ content in the 0–45 cm soil layer of the loam soil (**a**) and sandy loam soil (**b**). R—loam soil; S—sandy loam soil; L, M, and H—low, medium, and high rates of Cl application, respectively. ** indicates a highly significant correlation between rainfall and Cl^−^ content between the Cl treatments (*p* < 0.01).

The Cl^−^ dosage had a significant effect on the Cl^−^ leaching efficiency in the loam and sandy loam soils (Figure 5). The residual Cl^−^ content in the 0–45 cm soil layer was significantly correlated with rainfall despite the different chlorination treatments. However, the increases in the correlation coefficient and regression coefficients were consistent with the Cl^−^ dosage, which indicated that the leaching efficiency increased with increasing Cl^−^ application rate. Until the potato harvest stage, the Cl^−^ residual rates in the 0–45 cm soil layers for the loam and sandy loam soils were only 4–8.3% and 1.8–5%, respectively, and there were no significant differences in the Cl^−^ contents among the soils regardless of the treatment (*p* > 0.05).

**Figure 6:**
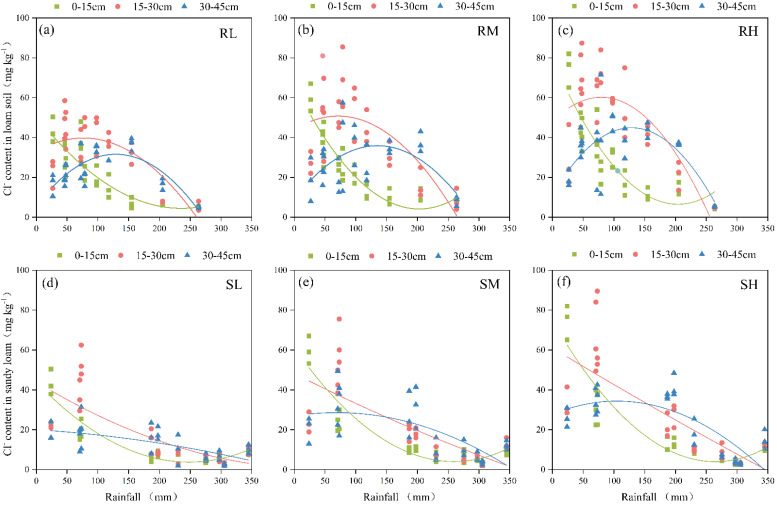
The effects of rainfall on the Cl^−^ content in loam soil (**a**–**c**) and sandy loam soil (**d**–**f**). R-loam soil; S—sandy loam soil; L, M, and H—low, medium, and high rates of Cl application, respectively.

**Figure 7:**
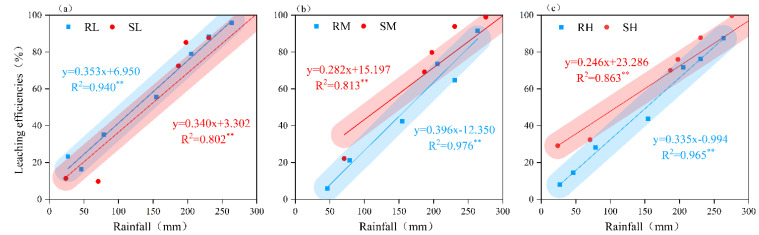
Critical rainfall for different leaching efficiencies of Cl^−^. (**a**–**c**)—low, medium, and high rates of Cl application in loam soil and sandy loam soil; R—loam soil; S—sandy loam soil; L, M, and H—low, medium, and high rates of Cl application. ** indicates highly significant correlation between rainfall and leaching efficiency (*p* < 0.01).

Rainfall was significantly negatively correlated (*p* < 0.01) with the Cl^−^ content in different soil layers for the loam and sandy loam soils (except for the 30–45 cm layer of the loam soil), but the correlation coefficients gradually decreased with increasing soil depth and Cl^−^ application rate (Table 1, Figure 6). The amount of rainfall was highly significantly positively correlated (*p* < 0.01) with the leaching efficiency of Cl^−^ in the loam and sandy loam soils (Figure 7). To achieve 30% Cl^−^ leaching efficiency in the 0–45 cm soil layer, 65–106 mm of rainfall was needed for the loam soil, whereas 27–79 mm of rainfall was needed for the sandy loam soil. When the Cl^−^ leaching efficiency was less than 30%, the loam soil required 1.5–1.7 times more water than the sandy loam soil at the same leaching efficiency. However, the difference in the rainfall required between the loam soil and sandy loam soil gradually decreased with increasing leaching efficiency, and there was no difference when the leaching efficiency reached 80%. The effects of the Cl^−^ dosage and soil texture on the Cl^−^ leaching efficiency gradually decreased with increasing rainfall.

The Cl^−^ content in the 0–15 cm soil layer exhibited highly significant (*p* < 0.01) responses to the soil texture (T), chlorine application rate (Cl), rainfall (R), and their interaction effects (Table 2). In contrast, the Cl^−^ contents in both the 15–30 cm and the 30–45 cm soil layers were significantly (*p* < 0.01) affected only by the chlorine application rate (Cl), rainfall amount (R), and their interactions. In addition, the Cl^−^ content in the 0–45 cm soil layer exhibited no significant (*p* > 0.01) responses to SO_4_^2−^- content in the 0–45 cm soil layer. The analysis of covariance (ANCOVA) (Table 3) demonstrates that, after controlling for the influence of sulfate (SO_4_^2−^), SO_4_^2−^ itself exhibited no significant effect on Cl^−^ (all soil layers: *p* > 0.05), confirming its nonsignificant results in the original ANOVA. The main effects of Cl application rate (Cl) and rainfall (R) remained significant (*p* < 0.01), with effect sizes (F-values) nearly identical to those without SO_4_^2−^ adjustment. Soil texture (T) showed significance only in the 0–15 cm layer, consistent with the original analysis, further supporting its surface-specific influence. Interaction terms (e.g., T × Cl, Cl × R) retained their significance after covariate inclusion, indicating that SO_4_^2−^ does not participate in Cl^−^ dynamics regulation.

### 2.4. Validation of Model with Field Experiments

**Figure 8:**
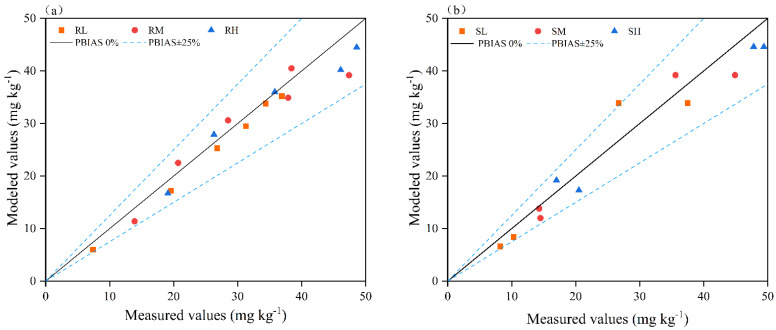
PBIAS (percentage bias) of Cl^−^ percentage deviation between measured and model-calculated values in loam soil (**a**) and sandy loam soil (**b**). R—loam soil; S—sandy loam soil; L, M, and H—low, medium, and high rates of Cl application, respectively.

The data measured in the field from 2022 to 2023 were input into the established model to generate estimated values of the soil Cl^−^ content. A comparison of the measured and estimated values revealed the good performance of the developed model, with all correlation coefficients (r) exceeding 0.8, coefficients of determination (R^2^) greater than 0.75, and normalized root mean square errors (NRMSEs) below 0.5 (Table 4). These results indicated an excellent goodness-of-fit between the actual Cl^−^ content and model-calculated values for both the loam and sandy loam soils (Figure 8). Comprehensive evaluation on the basis of multiple metrics confirmed the model’s high predictive accuracy, demonstrating its reliability for estimating Cl^−^ residue levels in these soil types.

### 2.5. Influence of Basal Application of KCl on Potato Tuber Yield

**Figure 9:**
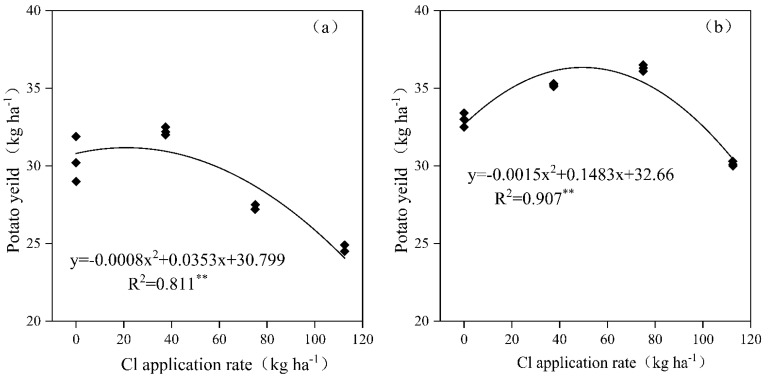
Influence of basal application of KCl on potato tuber yield in 2022–2023. Potato tuber yield data = average of two years. (**a**) R—loam soil; (**b**) S—sandy loam soil. ** indicates a highly significant correlation between Cl application rate and potato yield (*p* < 0.01).

The relative yield of potato refers to the ratio of yield under Cl application treatment to that under CK treatment. When the relative yield of potato tubers is less than 100%, it indicates yield suppression. A relative yield of 95–100% is considered the safe chloride application range, 80–90% indicates mild toxicity, and below 50% signifies severe toxicity. After basal KCl application in the loam soil, the total yield of potato tubers initially increased and then decreased with increasing Cl application rate, exhibiting a highly significant correlation (*p* < 0.01) (Figure 9a). When the Cl application rate was below 51.6 kg ha^−1^ (equivalent to 115 kg KCl ha^−1^), the relative yield exceeded 100%, indicating no yield suppression. At Cl application rates of 51.6–73.8 kg ha^−1^ (115–164 kg KCl ha^−1^), the relative yield ranged from 95% to 100%, falling within the safe Cl application range. In the sandy loam soil (Figure 9b), basal KCl application followed a similar trend, showing a highly significant correlation (*p* < 0.01). The relative yield reached 100% at a Cl application rate of 96.3 kg ha^−1^ (214 kg KCl ha^−1^), while a relative yield of 95% corresponded to 106.8 kg ha^−1^ (237 kg KCl ha^−1^).

## 3. Discussion

### 3.1. Effects of Rainfall on Exogenous Cl^−^ Transport and Leaching in Soils with Varying Textures

As a nonreactive ion, Cl^−^ is only weakly adsorbed into soil particle surfaces through minimal electrostatic interactions with limited positive charges, and soil water movement significantly influences Cl^−^ migration in soils [18,19]. Our results demonstrate that rainfall significantly affects Cl^−^ migration and leaching in soils, with its influence decreasing as soil depth increases. With the different chlorine application treatments, the Cl^−^ content in each soil layer exhibited a significant negative correlation with rainfall amount (*p* < 0.05). A rainfall amount of 200 mm was needed to completely translocate Cl^−^ from the 0–15 cm layer to the 30–45 cm layer in the loam soil, whereas only 150 mm of rainfall was needed for sandy loam, indicating slower Cl^−^ migration rates in loam than in sandy loam. When the rainfall reached 270 mm, no statistically significant differences (*p* > 0.05) in Cl^−^ content were detected between the soils with the chlorine-amended and unamended treatments across all the soil layers in either soil type (Figure 4). In the 0–45 cm soil layer, the Cl^−^ residual rate was only 8.7–14.9%, with the majority of Cl^−^ being distributed below 45 cm, a finding that is consistent with the results reported by Su et al. [20]. The loose structures of the surface soils combined with root distribution facilitate preferential flow, significantly increasing moisture infiltration and transport capacity in the upper soil layers [21,22]; this effect diminishes with increasing soil depth, resulting in progressively slower Cl^−^ migration rates across the soil profile [23].

Furthermore, under equivalent rainfall inputs, the Cl^−^ leaching efficiency increased with increasing chlorine application rates across all soil textures (Figure 5). The application of chlorine-containing fertilizer initially resulted in significant increases in Cl^−^ concentrations in the surface soils (Figure 1 and Figure 2); for example, when the rainfall was 20 mm, the Cl^−^ content in the 0–15 cm soil layer under chlorine application treatments was significantly higher than that under the non-chlorine treatment, and among the chlorine application treatments, the Cl^−^ content showed an order of Cl_H_ > Cl_M_ > Cl_L_. However, owing to the limited number of adsorption sites for Cl^−^ on soil particles, this phenomenon resulted in the increased leaching of free Cl^−^ during soil water migration in the case of high-chlorine treatments [24]. Analysis of the soil Cl^−^ content across potato growth stages revealed that the differential effects of chlorine application rates on leaching efficiency gradually diminished and ultimately disappeared as the growing season progressed. Postharvest analysis revealed that the Cl^−^ residual rates were less than 10% across soils, regardless of the soil texture or chlorine application treatment. This phenomenon occurred because, in addition to leaching, crop uptake of Cl^−^ helps maintain soil Cl^−^ equilibrium [23], ultimately resulting in a constant residual Cl^−^ level independent of the application rate. This finding aligns with the results reported by Mao et al. [25].

Furthermore, our results revealed that soil texture only significantly influenced the Cl^−^ leaching efficiency during the initial leaching phase (Figure 6 and Figure 7). When the leaching efficiency reached 30% and 50%, the loam soil required 50.9%–70.2% and 9.0%–13.7% more rainfall than the sandy loam soil, respectively. When the leaching efficiency reached 80%, no statistically significant difference (*p* > 0.05) in the required rainfall was detected between the loam and sandy loam soils. The results of this study align with those reported by Reynolds et al. [26]. To achieve equivalent leaching efficiency, loam soils require higher rainfall/irrigation amounts than sandy loam soils do (Figure 6). Under identical irrigation conditions, the Cl^−^ leaching efficiency in clay loam is lower than that in silty loam [27]. This phenomenon may be attributed to the low vegetation coverage during the initial experimental phase, where bare soil conditions render infiltration processes predominantly controlled by the soil pore structure [28,29]. The higher hydraulic conductivity of sandy loam results in greater initial leaching efficiency than that of loam. Consistent with Helle et al. [30], clay-rich soils provide more adsorption sites for Cl^−^, resulting in a greater Cl^−^ retention capacity. Sandy loam, which is characterized by higher porosity and macroporosity [31], tends to develop preferential flow paths, whereas in loam—with its dominant fine pores and charged aggregates—Cl^−^ transport is delayed due to the complexity of pore networks and increased adsorption of ions [32]. However, the influence of soil texture on leaching efficiency gradually diminished with increasing cumulative rainfall. When the cumulative rainfall reached 270 mm, no significant difference (*p* > 0.05) in Cl^−^ leaching efficiency was detected between the loam and sandy loam soils. This convergence likely occurred because, during the mid-to-late rainy season, sustained rainfall significantly alters the root-zone hydrodynamic conditions despite the increased potato canopy coverage. The high-intensity infiltration flux causes the Cl^−^ transport rates to become similar in both soil types, ultimately mitigating texture-dependent effects.

Our study, through theoretical analysis and field experiments, demonstrates that under these experimental conditions, sulfate (SO_4_^2−^) does not significantly influence the migration and leaching of chloride (Cl^−^) (Figure 1 and Figure 3). The cumulative leaching loss of Cl^−^ and the rainfall required for leaching showed no statistically significant differences with varying SO_4_^2−^ application rates (*p* > 0.05), as reinforced by the ANCOVA outcomes (Table 3). We attribute this observation to several key factors. First, the soils in Suihua and Mudanjiang are predominantly acidic, wherein Cl^−^ exhibits negligible adsorption to soil colloids, while SO_4_^2−^ undergoes specific adsorption with Fe/Al oxides [33]. Suihua’s black soil, characterized by high organic matter content, further ensures that SO_4_^2−^ preferentially occupies adsorption sites [34]. Although high concentrations of SO_4_^2−^ could theoretically enhance Cl^−^ migration by increasing ionic strength, the actual ionic strength variation induced by fertilization in this study likely only exerted a minor promoting effect on Cl^−^ transport. Additionally, in the aerobic rhizosphere environment, the immobilization of Cl^−^ by sulfate-reducing bacteria (SRB) is negligible [35]. Furthermore, when water percolates through soil pores, it transports both SO_4_^2−^ and Cl^−^ downward. These ions predominantly exist as independent species in the soil solution and do not chemically interact to form precipitates or complexes that could alter their leaching behavior [36]. Their relative positions and mobility in the soil solution remain largely unaffected by each other. Moreover, due to differences in ionic properties—such as radius, charge, and hydration energy—their migration resistance in soil pores varies significantly. Cl^−^, with its smaller ionic radius, lower charge, and weaker hydration, encounters minimal hindrance during pore-scale transport. In contrast, SO_4_^2−^, with its larger radius, higher charge, and stronger hydration, experiences substantially greater migration resistance [37]. Consequently, SO_4_^2−^ is unlikely to interfere with the leaching dynamics of Cl^−^.

### 3.2. Validation of Cl^−^ Retention Estimation Model

Model-based estimation serves as an efficient and rapid method for determining soil nutrient content, playing a critical role in guiding agricultural production and ecological and environmental research. To develop effective models, it is essential to ensure the validity of the parameters while considering the feasibility of acquiring these parameters, thereby guaranteeing the practical applicability of the developed model [38]. Through preliminary simulation experiments, our research team identified the rainfall amount, chloride application rate, and soil clay content as the dominant factors influencing soil Cl^−^ retention. On the basis of these findings, we developed an estimation model for residual soil Cl^−^ concentrations; specific information about this model can be found in the literature, such as in the study by Sun et al. [6], so it will not be discussed in detail in this article. An evaluation of model goodness-of-fit using measured versus estimated Cl^−^ concentrations in loam and sandy loam soils demonstrated the high predictive accuracy (R^2^ > 0.9) of the proposed model, confirming the reliability of the model for estimating residual Cl^−^ levels in these soil types (Figure 7, Table 4). This approach enables the following: (1) the determination of optimal irrigation volumes on the basis of target soil Cl^−^ contents and chloride application rates; (2) the calculation of appropriate chloride fertilizer doses according to target soil Cl^−^ levels and rainfall amounts. These functionalities provide a practical tool for agricultural decision-making regarding chloride fertilization and water management. (Note: Soil Cl^−^ thresholds for different potato growth stages are addressed in a subsequent study.)

### 3.3. Response of Cl^−^ Transport Dynamics to Potato Growth Stages and Tuber Yield

**Figure 10:**
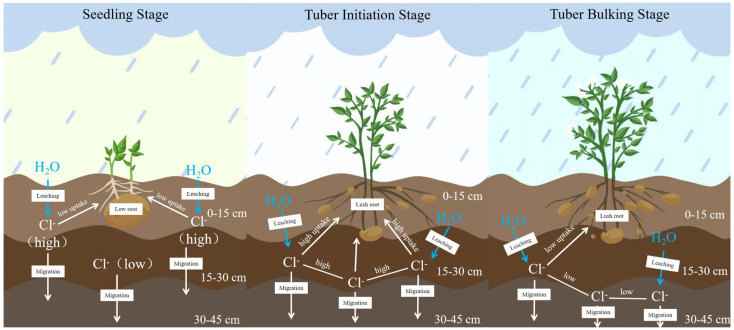
Dynamics of Cl^−^ transport with rainfall during different fertility periods in potato crops.

In this study, the effects of natural rainfall on Cl^−^ transport and distribution in soils undergoing potato cultivation were investigated by comprehensively considering rainfall interception by the vegetation canopy and reduced leaching volume due to soil water uptake by plants. Rainfall data from Suihua and Mudanjiang over the past 20 years (May–September) indicate that cumulative rainfall during the potato sowing-to-emergence period has consistently exceeded 30 mm. During this stage, the applied Cl^−^ primarily remains within the 0–15 cm fertilizer application layer. However, owing to the limited root system and low nutrient uptake capacity of potato plants at this growth stage, Cl^−^ absorption by potatoes is relatively low. At both experimental sites, the cumulative rainfall during the seedling to tuber initiation stages ranged from 70 to 130 mm. During this period, Cl^−^ migrated downward with increasing soil moisture and primarily accumulated in the 15–30 cm soil layer (Figure 10). At this growth stage, the well-developed potato root system, with its expanded absorption zone coinciding with the Cl^−^-enriched soil layer (15–30 cm), was associated with peak Cl^−^ uptake by potato plants. Maintaining soil Cl^−^ concentrations below the safety threshold during this stage is critical. As the potatoes entered the tuber bulking phase, the cumulative rainfall at both sites exceeded 200 mm, resulting in predominant Cl^−^ accumulation in the 30–45 cm soil layer. As shallow-rooted crops [39,40], potato plants predominantly develop root systems within the upper 30 cm of soil [41,42]. Consequently, the probability of Cl^−^ uptake by roots decreased significantly. When the cumulative rainfall reached 270 mm, no significant difference (*p* > 0.05) in Cl^−^ content was detected between the chlorinated and non-chlorinated soils across the corresponding soil layers.

Our results demonstrated that when cumulative rainfall reached 270–300 mm after fertilization, KCl application rates ≤ 250 kg ha^−1^ did not cause Cl^−^ accumulation in the primary potato root zone (0–30 cm soil layer), thereby preventing adverse effects on subsequent crops. However, excessive chlorine application negatively impacted tuber yield. By establishing regression equations between the relative yield of crops under different treatments and plant nutrient content, the corresponding nutrient concentrations at relative yields of 95%, 90%, and 80% were calculated using these regression equations to determine critical nutrient thresholds for the crop [43,44,45]. In loam soil, the safe application range for KCl was determined to be 115–164 kg ha^−1^, while in the sandy loam soil, the safe KCl application range was 214–237 kg ha^−1^ (Figure 8). Nie et al. [46] demonstrated through field experiments that the leaf Cl^−^ content during the potato seedling and tuber initiation stages is significantly correlated (*p* < 0.05) with tuber yield. During these growth stages, basally applied Cl^−^ primarily accumulates in the 15–30 cm soil layer, which coincides with the primary root distribution zone. Based on the analysis of chlorine leaching characteristics in soil, under conditions of low early-season rainfall, chlorine mobility was significantly influenced by soil texture. Loam soils retained higher chloride ion concentrations in the plow layer, which restricted plant uptake and consequently reduced potato yield. Therefore, KCl application rates during basal fertilization should be carefully controlled in loam soils, whereas higher KCl application rates can be utilized in sandy loam soils. These findings highlight the need to optimize Cl^−^ application rates according to regional rainfall patterns to maintain soil Cl^−^ concentrations below established safety thresholds throughout these critical growth phases.

## 4. Materials and Methods

### 4.1. Site Characteristics and Experimental Materials

Two years of field experiments were conducted in 2022 and 2023 in Suihua city (127°06′ N, 46°60′ E) and Mudanjiang city (129°50′ N, 44°43′ E), Heilongjiang Province, China, with the two sites both being experimental fields in a science and technology park. The tested potato variety was ‘Youjin’, a major early-maturing cultivar in Heilongjiang Province, with a growth period of approximately 65–70 days (May–September each year). Both regions have a temperate monsoon climate with cold and dry winters but warm and rainy summers. The rainy season mainly occurs from May to September. According to the data statistics from the China Meteorological Administration, the average rainfall levels from May to September over the past 20 years were 368 mm in Suihua and 340 mm in Mudanjiang. The weather stations were positioned 200 m from the Suihua experimental field and approximately 50 m away from the Mudanjiang experimental field. Daily rainfall measurements (mm) and precipitation timing (start/end time) were recorded. All sensors complied with WMO (World Meteorological Organization) standards. China has not yet established a universally adopted unified soil texture classification system. The current classification standards mainly follow the international system. With reference to the International Standard Soil Texture Classification (ISSS) [47], the soil texture of the Suihua field can be classified as loam soil, whereas that of the Mudanjiang field can be classified as sandy loam soil (Table 5 and Appendix A Table A1). Over the two-year study period, the water-soluble Cl^−^ content in the soil at both experimental sites ranged from 8.6 to 12.5 mg kg^−1^ (before sowing).

### 4.2. Cl Treatment and Application

The experiment utilized a randomized block design; each plot consisted of 6 ridges, with each ridge measuring 12 m in length and 0.8 m in width, resulting in a total plot area of 57.6 m^2^. The potato plants were spaced approximately 25 cm apart within the ridges. All the treatments were conducted in triplicate (three replicates). The experimental treatments remained consistent across both test sites.

The fertilizers used in the experiment included urea (N 46%), DAP (N 18%, P_2_O_5_ 46%), K_2_SO_4_ (K_2_O 50%), and KCl (K_2_O 60%, Cl 45%). N (200 kg ha^−1^) was applied in two equal splits (basal and topdressing). The basal N was supplied by urea and DAP, while the topdressing N was provided solely by urea. P_2_O_5_ (90 kg ha^−1^) was applied as a one-time basal dose, supplied by DAP. K_2_O (300 kg ha^−1^) was split equally between the basal and topdressing applications, provided by K_2_SO_4_ and KCl. For the basal fertilizer, three Cl application rates were established: 37.5 kg ha^−1^ (L, 83 kg KCl ha^−1^, 20 mg kg^−1^), 75 kg ha^−1^ (M, 167 kg KCl ha^−1^, 40 mg kg^−1^), and 112.5 kg ha^−1^ (H, 250 kg KCl ha^−1^, 60 mg kg^−1^). To maintain consistent K_2_O application rates, the remaining potassium requirement in the Cl-treated plots was supplemented with potassium sulfate. The control (CK) treatment exclusively used potassium sulfate for both the basal and topdressing applications (Table 6). The experimental setups were identical for both the loam (R) and sandy loam (S) soil treatments.

### 4.3. Field Management

In Suihua, the potatoes were sown on 5 May 2022. Topdressing was applied on 22 June. The potatoes were harvested for yield measurement on 2 September. In 2023, sowing occurred on 5 May, topdressing occurred on 17 June, and harvesting occurred on 6 September. In Mudanjiang, the potatoes were sown on 2 May 2022, topdressing occurred on 21 June, and harvesting occurred on 21 August. In 2023, sowing occurred on 3 May, topdressing occurred on 13 June, and harvesting occurred on 12 September.

In the Suihua experimental site, the preceding crops were maize in both 2022 and 2023, while the Mudanjiang site had maize and soybean as preceding crops in the respective years. The seed potatoes were treated with wood ash for disease prevention prior to planting. At both experimental sites, conventional fungicides were applied weekly during the tuber initiation stage to control weeds, late blight (*Phytophthora infestans*), and common scab (*Streptomyces scabies*). Spraying continued until the starch accumulation stage of potato growth.

The basal fertilizer was applied using a mechanical planter(Ningbo Hengtai Benye Agricultural Equipment Co., Ltd., Ningbo, China), which simultaneously placed fertilizer and seed potatoes at sowing depths of 10–15 cm. The fertilizer was positioned below the seed potatoes. Topdressing was performed when 90% of the potato seedlings had emerged and was applied to both sides of the ridges during inter-row cultivation and hilling.

### 4.4. Soil Sample Collection

Prior to fertilizer application, the baseline fertility of the soil was determined using a 7-point sampling method across different soil layers at both locations. Following KCl application, soil samples were collected by randomly selecting 5 sampling points from the potato ridges during each sampling event. At each point, stainless steel soil augers were used to collect samples from three layers: 0–15 cm, 15–30 cm, and 30–45 cm. The samples from each layer were thoroughly homogenized, and the quartering method was employed to reduce the sample size to approximately 1000 g for laboratory analysis. The samples were then air-dried in a shaded environment, ground, and passed through a 1 mm sieve for subsequent soil Cl^−^ content determination.

In Suihua, soil samples were collected at 40, 48, 53, 72, and 94 DAS (days after sowing) in 2022 and at 19, 38, 56, 76, and 98 DAS in 2023. In Mudanjiang, soil samples were collected at 43, 63, 70, 79, and 99 DAS in 2022 and at 20, 41, 62, 82, and 102 DAS in 2023.

### 4.5. Soil Test Methods

The soil characteristics were determined according to Bao’s method [48]. Soil organic matter, available N, P, K, and SO_4_^2−^, and pH value were determined by wet oxidation, the alkali-diffusion method, the Olsen method, the NH_4_OAc extraction–flame photometry method, BaSO_4_ turbidimetry, and a pH meter (water/soil was 2.5:1). The water-soluble Cl^−^ content was determined as follows: 10 g of a soil sample sieved at 1 mm (accuracy: 0.001 g) was precisely weighed, 50 mL of deionized water was added, the mixture was shaken at 200 rpm for 3 min for extraction, the suspension was filtered, and the Cl^−^ concentration in the filtrate was measured by ISE (CLS-10A portable chloride analyzer, Haiheng Electric and Instrument Co., Ltd., Shanghai, China). The soil bulk density was determined by the core method [49]. The soil clay, silt, and sand content was measured using a Topsizer Plus laser (Zhuhai OMEC Instruments Co., Ltd., Zhuhai, China) particle size analyzer [50].

### 4.6. Index Calculation

The following are the formulas for calculating Cl-related deformation:

$${\mathrm{Cl}\;\mathrm{accumulation}\;\mathrm{amount}\;(\mathrm{ClA},\;\mathrm{kg}\;\mathrm{ha}}^{-1}{)=C}_{T}\times \mathrm{BD}\times D$$

where C_T_ represents the concentration of Cl^−^ in the soil (mg kg^−1^), BD represents the bulk density (g cm^−3^), and D represents the soil depth (cm).

$$\mathrm{Cl}\;\mathrm{leaching}\;\mathrm{factor}\;(\mathrm{ClLF},\;\%)=\frac{{\mathrm{Cl}}_{r}-\left({\mathrm{ClA}}_{T}{-\mathrm{ClA}}_{\mathrm{CK}}\right)}{{\mathrm{Cl}}_{r}}\times 100\%$$

where Cl_r_ (kg ha^−1^) represents the amount of Cl^−^ applied and ClA_T_ (kg ha^−1^) and ClA_CK_ (kg ha^−1^) represent the accumulation amounts of Cl^−^ with and without Cl application, respectively.

Cl residual ratio (ClRR, %)=1−ClLF-ClP

where ClLF (%) represents the Cl leaching factor and ClP (%) represents the potato plant Cl absorption rate.

### 4.7. Model Validation Indices

Through preliminary simulation experiments, our research team identified the rainfall amount, chloride application rate, and soil clay content as the dominant factors influencing soil Cl^−^ retention. On the basis of these findings, we developed an estimation model for residual soil Cl^−^ concentrations. The current study focused exclusively on the field validation of the pre-existing model, with full developmental details available in [6]. The conformity of the Cl^−^ content between the measured values and model estimations was evaluated on the basis of Pearson’s correlation coefficient (r), the coefficient of determination (R^2^), the normalized root mean square error (NRMSE), and the percentage bias (PBIAS) to verify whether it could be applied to actual agricultural production. The system for evaluating model performance is presented in Table 7; the R^2^, NRMSE, and PBIAS score ranges were based on the standards provided by Moriasi et al. [51] and Serban et al. [52].

$$Y=38.518-0{.219X}_{1}+0{.143X}_{2}+0{.557X}_{3}$$

In the Cl^−^ residual estimation model, X_1_ is the rainfall (mm), X_2_ is the chlorine application rate (kg ha^−1^), and X_3_ is the soil clay content (%).

$$R^{2}=1-\frac{\sum_{i}(Y_{\mathrm{iMO}}-Y_{\mathrm{iME}}{)}^{2}}{\sum_{i}({\bar{Y}}_{\mathrm{iME}}-Y_{\mathrm{iME}}{)}^{2}}$$

where R^2^ is the coefficient of determination, Y_iME_ is the i-th measured value and Y_iMO_ is the i-th model-calculated value.

$$\mathrm{RMSE}=\sqrt{\frac{\sum_{i=1}^{n}(Y_{\mathrm{iME}}-Y_{\mathrm{iMO}}{)}^{2}}{n}}$$

where RMSE is the root mean square error, Y_iME_ (mg kg^−1^) is the i-th measured value, Y_iMO_ (mg kg^−1^) is the i-th model-calculated value, and n is the number of data points.

$$\mathrm{NRSME}=\frac{\mathrm{RSME}}{Y_{\mathrm{ME}-\mathrm{MAX}}-Y_{\mathrm{ME}-\mathrm{MIN}}}$$

where NRMSE is the normalized root mean square error, Y_ME−MAX_ (mg kg^−1^) is the maximum observed value, and Y_ME−MIN_ (mg kg^−1^) is the minimum observed value.

$$\mathrm{PBIAS}=\frac{\sum_{i=1}^{n}Y_{\mathrm{iME}}-Y_{\mathrm{iMO}}}{\sum_{i=1}^{n}Y_{\mathrm{iME}}}\times 100$$

where PBIAS is the percentage bias (%), Y_iME_ (mg kg^−1^) is the i-th measured value, and Y_iMO_ (mg kg^−1^) is the i-th model-calculated value.

### 4.8. Statistical Analysis

Assuming that all the data followed a normal distribution and passed homogeneity of variance tests, statistical analyses were performed using SPSS 21.0. One-way ANOVA and Duncan’s multiple range test (MRT) (*p* < 0.05) were applied to determine significant differences in the Cl^−^ contents across the soil layers in the soils treated with different chlorine applications. Pearson’s correlation coefficient was used to assess the relationships between the Cl^−^ content and rainfall in soils across different treatments and soil layers (*p* < 0.01). When the F test results were statistically significant at α = 0.01, indicating that rainfall, the chlorine application rate, and soil texture had significant effects on the Cl^−^ content in different soil layers, two-way ANOVA (*p* < 0.01) was used to validate the influences of these factors. Origin 2021 was used for data visualization, and simple linear regression analysis was performed to quantify the relationship between rainfall and Cl^−^ leaching efficiency under different chlorine application treatments. The model validation results were computed using the previously provided equations.

## 5. Conclusions

The validation analysis demonstrated that the multifactor regression model (Y = 38.518 − 0.219X_1_ [rainfall] + 0.143X_2_ [chloride application rate] + 0.557X_3_ [clay content]) accurately estimated Cl^−^ retention under varying conditions. The validation results revealed an excellent goodness-of-fit (r/R^2^ > 0.9, NRMSE < 0.1), confirming the superior performance of the proposed model. The results revealed that exogenous Cl^−^ transport in the loam and sandy loam soils was coregulated by three factors: (1) rainfall amount, (2) chloride application rate, (3) soil texture. The influence of soil texture dynamically varied with increasing rainfall and became negligible when the cumulative rainfall amount reached ≥270 mm (*p* > 0.05 between soils with different textures). On the basis of these findings, a model was established as a quantitative decision-making tool for precise chloride management in rain-fed potato production systems. Furthermore, in potato growing seasons with natural rainfall amounts ≥ 300 mm, maintaining KCl application rates at 80–250 kg ha^−1^ (equivalent to 37.5–112.5 kg Cl ha^−1^) in loam and sandy loam soils effectively prevents Cl^−^ accumulation in the primary root zone (0–30 cm soil layer). In loam soil, the safe application range for KCl was determined to be 115–164 kg ha^−1^, while in sandy loam soil, the safe KCl application range was 214–237 kg ha^−1^.

## Figures and Tables

**Table 1:** Fitting equations between rainfall and Cl^−^ content in different soil layers (*p* < 0.01).

Treatment	Soil Depth (cm)	Loam Soil (R)		Sandy Loam Soil (S)	
		Regression Equation	r	Regression Equation	r
Cl_L_	0–15	y = (4.55 × 10^−4^)x^2^ − 0.27x + 41.93	−0.823 **	y = (6.55 × 10^−4^)x^2^ − 0.32x + 44.09	−0.785 **
	15–30	y = (8.31 × 10^−4^)x^2^ − 0.39x + 50.70	−0.704 **	y = (2.04 × 10^−4^)x^2^ − 0.19x + 44.10	−0.755 **
	30–45	y = (−7.68 × 10^−4^)x^2^ + 0.16x + 15.55	−0.318	y = (−7.25 × 10^−5^)x^2^ − 0.02x + 20.03	−0.650 **
Cl_M_	0–15	y = 0.00151x^2^ − 0.61x + 66.42	−0.800 **	y = (8.49 × 10^−4^)x^2^ − 0.44x + 61.21	−0.829 **
	15–30	y = (−0.00128)x^2^ + 0.32x + 8.55	−0.650 **	y = (6.68 × 10^−5^)x^2^ − 0.16x + 48.25	−0.750 **
	30–45	y = −0.00137x^2^ + 0.35x + 11.42	−0.342	y = (−3.47 × 10^−4^)x^2^ + 0.05x + 26.95	−0.636 **
Cl_H_	0–15	y= 0.00108x^2^ − 0.49x + 64.34	−0.727 **	y = 0.00101x^2^ − 0.53x + 74.73	−0.836 **
	15–30	y= −0.00193x^2^ + 0.31x + 47.98	−0.644 **	y = (2.94 × 10^−5^)x^2^ − 0.19x + 61.06	−0.793 **
	30–45	y= −0.00204x^2^ + 0.52x + 11.19	−0.131	y = (−6.03 × 10^−4^)x^2^ + 0.13x + 27.72	−0.674 **

**Note:** Cl_L_, Cl_M_, Cl_H_—low, medium, and high rates of Cl application; x—rainfall; y—Cl^−^ content. ** indicates highly significant correlation between rainfall and Cl^−^ content (*p* < 0.01).

**Table 2:** ANOVA for Cl^−^ content in different soil layers.

Sources of Variance	0–15 cm	15–30 cm	30–45 cm
SO_4_^2−^ content in soil (S)	0.739	0.386	0.057
Soil texture (T)	20.786 **	10.816	5.563
Cl application rate (Cl)	78.131 **	68.382 **	63.750 **
Rainfall (R)	43.950 **	30.998 **	15.979 **
T × Cl	3.495 **	1.083	0.861
T × R	2.946 **	0.562	0.014
Cl × R	5.718 **	4.545 **	2.104 **

**Note:** ** indicates a highly significant correlation between the factors and the Cl^−^ content in the different soil layers (*p* < 0.01).

**Table 3:** ANCOVA for Cl^−^ content in different soil layers with SO_4_^2−^ as covariate.

Sources of Variance	0–15 cm		15–30 cm		30–45 cm	
	F	*p*	F	*p*	F	*p*
SO_4_^2−^ content in soil (Covariate)	0.82	0.369	0.41	0.524	0.06	0.086
Soil Texture (T)	20.01 **	0.002	10.50	0.052	5.40	0.120
Cl application rate (Cl)	77.95 **	<0.001	68.20 **	<0.001	63.60 **	<0.001
Rainfall (R)	43.80 **	<0.001	30.90 **	<0.001	15.80 **	0.003
T × Cl	3.45 **	0.008	1.05	0.352	0.84	0.432
T × R	2.91 *	0.013	0.55	0.580	0.01	0.982
Cl × R	5.68 **	<0.001	4.51 **	0.002	2.08 **	0.098

**Note:** * (*p* < 0.05) and ** (*p* < 0.01) indicate highly significant correlations between the factors and the Cl^−^ content in the different soil layers (*p* < 0.05).

**Table 4:** Model validation results (*p* < 0.01).

Validation Metrics	Loam Soil (R)	Sandy Loam Soil (S)
r	0.976	0.969
R^2^	0.927	0.933
NRMSE	0.054	0.063
PBIAS	[−9.1%, 19.3%]	[−27.0%, 19.0%]

**Table 5:** Basic physical and chemical properties of soil.

Soil Index	2022		2023	
	Suihua	Mudanjiang	Suihua	Mudanjiang
Soil type	Black soil	Dark brown Soil	Black soil	Dark brown Soil
Soil texture	Loam soil	Sandy loam soil	Loam soil	Sandy loam soil
Organic matter (g kg^−1^)	31.8	22.9	36.4	24.7
Available N (mg kg^−1^)	138	69	158	78
Available P (mg kg^−1^)	12	16	27	22
Available K (mg kg^−1^)	206	168	215	173
pH	5.7	5.8	5.4	6.1
0–15 cm water-soluble Cl^−^ (mg kg^−1^)	12.5	8.6	10.8	10.6
15–30 cm water-soluble Cl^−^ (mg kg^−1^)	11.5	9.3	9.2	8.7
30–45 cm water-soluble Cl^−^ (mg kg^−1^)	10.9	7.9	11.0	9.7
SO_4_^2−^ (mg kg^−1^)	78.3	81.9	55.2	65.2
Bulk density (g cm^−3^)	1.32	1.36	1.31	1.38
Clay (%)	12.9	9.8	12.7	10.8
Silt (%)	37.9	34.1	36.4	31.4
Sand (%)	49.2	56.1	50.9	57.8

**Table 6:** Nutrient application programs for field experiments.

Treatment	Base Fertilizer (kg ha^−1^)					Dressing Fertilizer (kg ha^−1^)	
	N	P_2_O_5_	K_2_O (K_2_SO_4_)	K_2_O (KCl)	Cl	N	K_2_O (K_2_SO_4_)
CK	100	90	150	-	-	100	150
RL/SL	100	90	100	50	37.5	100	150
RM/SM	100	90	50	100	75	100	150
RH/SH	100	90	-	150	112.5	100	150

**Note:** CK—control treatment; R—loam soil; S—sandy loam soil; L, M, and H—low, medium, and high chlorine levels, respectively.

**Table 7:** Indicators to assess model fit and range of values (*p* < 0.01).

Norms	Optimum Value	Goodness-of-Fit Index (GFI)		
		Medium or Moderate	High	Very High or Extremely High
r	±1	|r| ≤ 0.6	0.6 < |r| ≤ 0.8	|r| > 0.8
R^2^	1	<0.65	0.65–0.75	>0.75
NRMSE	0	>0.7	0.5–0.7	<0.5
PBIAS	0	<−50% or >50%	−50% to −25% or 25% to 50%	−25% to 25%

## Data Availability

Data are contained within the article.

## Abbreviations

The following abbreviations are used in this manuscript:

RMSE	Root Mean Square Error
NRMSE	Normalized Root Mean Square Error
PBIAS	Percentage Bias
DAS	Days After Sowing
MRT	Multiple Range Test
ISE	Ion-Selective Electrode
ISSS	International Standard for Soil Texture Classification
SRB	Sulfate-Reducing Bacteria

## Appendix A

China has not yet established a universally adopted unified soil texture classification system. The current classification standards mainly follow the international system (ISSS). Table A1 presents the International Standard for Soil Texture Classification, which is characterized by (1) using clay content as the primary criterion, where soils with <15% clay are classified into sand and loam texture groups, those with 15–25% clay as clay loam texture group and those with >25% clay as clay texture group; (2) prefixing the texture group name with “silty-” (or “sandy-”) when the silt (or sand) content exceeds 45%; (3) applying the prefix “sandy-” to soils containing 55–85% sand, designating those with 85–90% sand as loamy sand, and classifying soils with >90% sand as sand [53].

**Table A1:** International Standard for Soil Texture Classification.

Texture Name		Particle Size Composition (%)		
		Clay (<0.002 mm)	Silt (0.002–0.02 mm)	Sand (0.02–2 mm)
Sandy soil	Sandy soil or loamy sand	0–15	0–15	85–100
Loam	Sandy loam	0–15	0–45	55–85
	Loam	0–15	30–45	40–55
	Silty loam	0–15	45–100	0–55
Clay loam	Sandy clay loam	15–25	0–30	55–85
	Clay loam	15–25	20–45	30–55
	Silty clay loam	15–25	45–85	0–40
Clay soil	Sandy clay	25–45	0–20	55–75
	Loamy clay	25–45	0–45	10–55
	Silty clay	25–45	45–75	0–30
	Clay	45–65	0–35	0–55
	Heavy clay	65–100	0–35	0–35
